# Supplementary material for: Hybrid Models and Biological Model Reduction with PyDSTool
Source: PLoS Comput Biol. 2012 Aug 9;8(8):e1002628. doi: 10.1371/journal.pcbi.1002628 (PMC3415397; doi:10.1371/journal.pcbi.1002628)
Supplement: Text S4 — Complete source code for the PyDSTool package (version 0.88.120504). Includes API documentation and help files linking to web pages. This file is identical to the current public release on Sourceforge.net. (ZIP) [file pcbi.1002628.s004.zip › PyDSTool/html/toc-PyDSTool.Interval'-module.html]

xml version="1.0" encoding="ascii"?


Interval'


# Module Interval'

---

## Classes

Interval  

IntervalMembership

## Functions

isinterval  
 issingleton  

## Variables

API

Continuous

Discrete

Inf

LargestInt32

MIN\_EXP

NaN

\_1DimplicitSolveMethods

\_all\_complex

\_all\_float

\_all\_int

\_all\_numpy\_complex

\_all\_numpy\_float

\_all\_numpy\_int

\_complex\_types

\_float\_types

\_implicitSolveMethods

\_int\_types

\_num\_equivtype

\_num\_maxmin

\_num\_name2equivtypes

\_num\_name2type

\_num\_type2name

\_num\_types

\_pytypefromtype

\_real\_types

\_seq\_types

c

contained  

isfinite

isinf

isnan

n

notcontained  

null\_predicate

re\_number

sign

targetLangs

u

uncertain  

---

[hide private]
